# Primary Adrenal Insufficiency (Addison’s Disease) Presenting as Sun Tan-Like Skin Pigmentation: A Case Report

**DOI:** 10.7759/cureus.49837

**Published:** 2023-12-02

**Authors:** Mohammed F Bondagji, Hamzah Qul, Ali Nahhas, Ebtesam S Allehaibi, Amal A Banjer, Ghadi A Alghamdi, Khalid Al Hawsawi

**Affiliations:** 1 Medicine and Surgery, College of Medicine, Umm Al-Qura University (UQU), Makkah, SAU; 2 Internal Medicine, Endocrinology Unit, King Abdulaziz Hospital, Makkah, SAU; 3 Internal Medicine, Neurology Unit, King Abdulaziz Hospital, Makkah, SAU; 4 Dermatology, King Abdulaziz Hospital, Makkah, SAU

**Keywords:** sun exposed, adrenal insufficiency, cutaneous, addison's disease, skin pigmentation

## Abstract

Primary adrenal insufficiency (PAI), also known as Addison's disease (AD), is a condition resulting from adrenal gland diseases that lead to glucocorticoid and/or mineralocorticoid deficiency, in addition to androgen deficiency in females. Here, we report a 40-year-old male indoor worker with an insignificant past medical history who presented to the dermatology clinic with a history of asymptomatic, slowly progressive skin hyperpigmentation for the past three years. It was associated with fatigue and weight loss. Skin examination revealed diffuse, non-scaly hyperpigmented patches on his face, dorsae of the hands, and palms. Early morning cortisol and adrenocorticotropic hormone (ACTH) serum levels were 1.00 µg/dl (5.0-19.4 µg/dl) and 2000 pg/mL (7.2-63.3 pg/mL), respectively. Based on the above clinical and laboratory findings, a diagnosis of AD was made. The patient was started on the following medications for 14 days: hydrocortisone 20 mg in divided doses (15 mg am/5 mg pm) and fludrocortisone 0.1 mg once daily (od). On the second visit, the patient's symptoms (both the cutaneous hyperpigmentation and fatigue) significantly improved, but he was complaining of edema in both upper and lower limbs, so the dose of fludrocortisone was reduced to 0.05 mg od.

## Introduction

Primary adrenal insufficiency (PAI), also known as Addison's disease (AD), is a condition of the adrenal glands that causes deficiencies in glucocorticoids and/or mineralocorticoids, as well as androgen deficiency in women. It has multifactorial etiologies; however, autoimmune causes for primary AD are more common in the Western world. The causes include autoimmune diseases, which are more common in the developing world, infections such as tuberculosis, disseminated fungal infections, syphilis, adrenal infarction caused by hemorrhage associated with septicemia (Waterhouse-Friderichsen syndrome) commonly related to pathogens like *Pseudomonas aeruginosa* and *Neisseria gonorrhoeae*, meningococcemia, adrenal vein thrombosis, blunt trauma to the adrenal gland, adrenal metastases, or drugs such as checkpoint inhibitors and inhibitors of cortisol biosynthesis [1]. The clinical features of adrenal insufficiency are often nonspecific, including fatigue, weight loss, gastrointestinal complaints such as nausea and vomiting, abdominal pain, and diarrhea that alternate with constipation, reproductive issues in women (due to the impact of weight loss and chronicity it will lead to amenorrhea), musculoskeletal symptoms such as diffuse myalgia and arthralgia, psychiatric symptoms, and auricular-cartilage calcification. The more specific features of PAI include postural hypotension, salt craving, mucocutaneous hyperpigmentation, loss of androgen-stimulated (axillary, pubic) hair in post-pubertal women, and fibrosis and calcification of cartilage, including the ear (rare) [2]. The cutaneous hyperpigmentation of AD is characterized by diffuse pigmentation that is accentuated in sun-exposed skin, prominent at sites of trauma. The pigmentation also appears in the axillae, perineum, nipples, palmar creases, mucous membranes, hair, and nails (longitudinal melanonychia). Patients may show an increased number of melanocytic nevi, freckles, and lentigines.

## Case presentation

**Figure 1:**
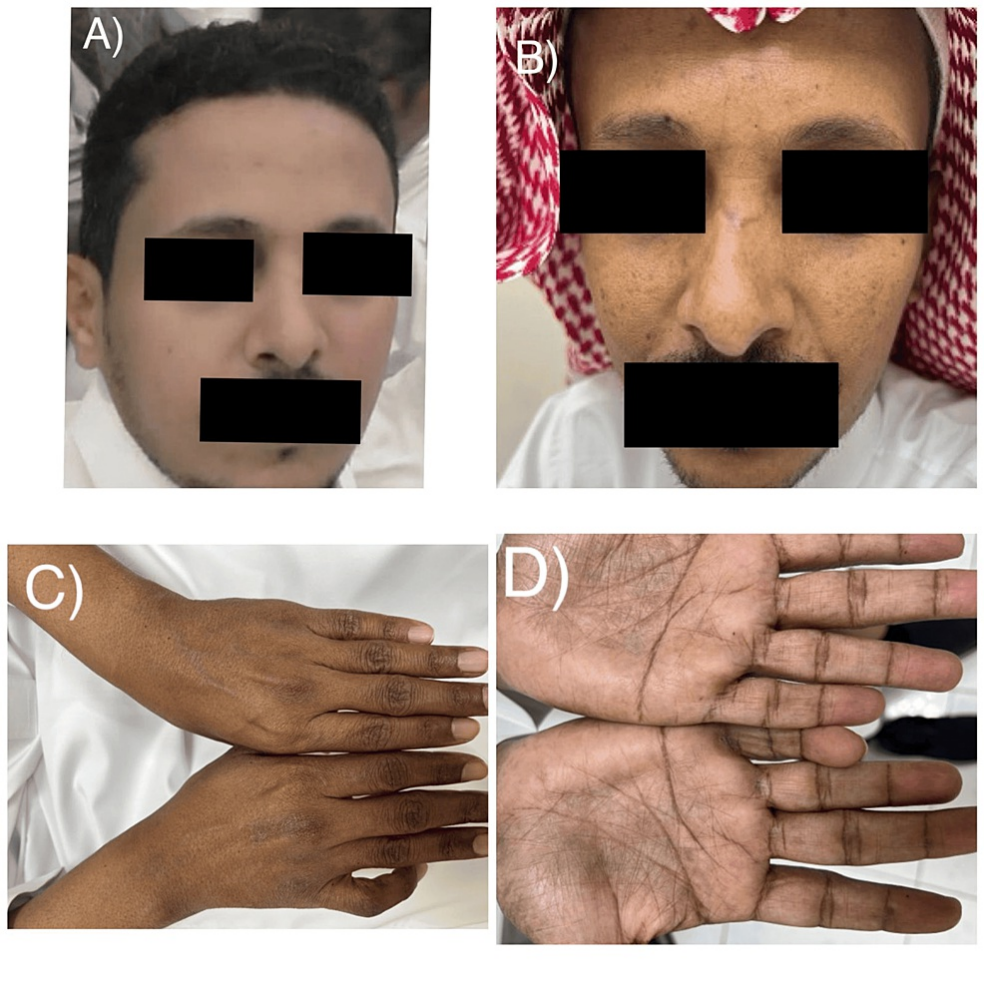
Skin examination showing the color of the face three years ago (A), and currently diffuse hyperpigmented patches on the face (B), dorsum of the hands with a sharp demarcation line between sun-exposed and sun-protected areas (C), and palms (D).

**Figure 2:**
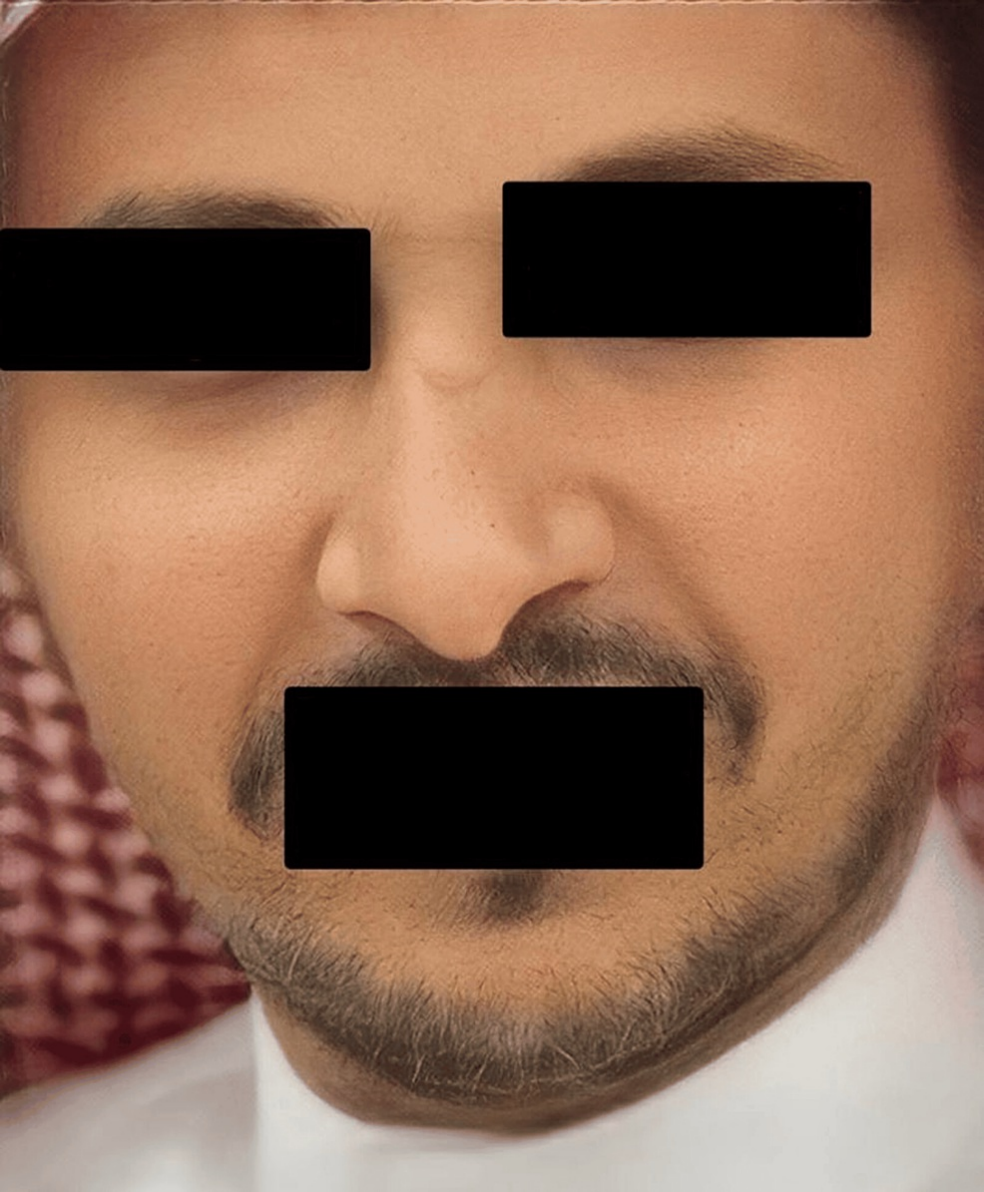
Cutaneous hyperpigmentation significantly improved two weeks after treatment.

A 40-year-old male with an insignificant past medical history presented to the dermatology clinic with a history of asymptomatic, slowly progressive skin pigmentation over the past three years. It was associated with fatigue, weight loss, and no history of excessive sun exposure. As an indoor worker, he had no complaints of photosensitivity, gastrointestinal issues, headache, dizziness, syncopal attacks, or salt craving, and he was not taking any medications. He had a family history of a similar condition (his brother was diagnosed with PAI associated with hypothyroidism: Schmidt syndrome). Skin examination revealed diffuse, non-scaly hyperpigmented patches on his face, dorsum of the hands, and palms (Figure 1). There was no mucous membrane or nail hyperpigmentation. Laboratory findings showed that complete blood count (CBC), hemoglobin, liver enzymes, sodium, potassium, chloride, urea, and creatinine were all within normal levels. Early morning cortisol and adrenocorticotropic hormone (ACTH) serum levels were 1.00 µg/dl (5.0-19.4 µg/dl) and 2000 pg/mL (7.2-63.3 pg/mL), respectively. No sizable adrenal masses could be detected by CT scan of the adrenals. Based on the above clinical and laboratory findings, a diagnosis of PAI was made. The patient was started on the following medications for 14 days: hydrocortisone 20 mg PO (divided dose 15 mg am/5 mg pm), fludrocortisone 0.1 mg PO od. On the second visit, the patient's symptoms (both the cutaneous hyperpigmentation and fatigue) significantly improved (Figure 2), but he was complaining of edema in both upper and lower limbs, so the dose of fludrocortisone was reduced to 0.05 mg PO od.

## Discussion

The damaged adrenal glands' reduced ability to synthesize cortisol leads to a reduction in the hypothalamus-pituitary axis negative feedback loop. As a result, there is an increase in corticotropin-releasing hormone and proopiomelanocortin synthesis. Proopiomelanocortin is a precursor to many physiologically active hormones, including melanocyte-stimulating hormone (MSH) and ACTH. Consequently, increased MSH levels cause epidermal melanocytes to start producing melanin [3].

The onset of AD can be chronic (similar to our patient) or acute (adrenal crisis with potentially fatal outcomes). Like our patient, the onset of AD can be very insidious, taking years, resulting in a delay in diagnosis. Although a wide variety of skin pigmentations is seen in AD, our patient exhibited a few of them, including diffuse pigmentation in sun-exposed skin (face and dorsae of hands) and palms. The differential diagnosis includes sun tan, AD, Cushing's disease, liver disease, renal failure, drug reactions, and vitamin B deficiency (B3, B9, B12).

The diagnosis of AD is very likely if the serum cortisol level is less than 140 nmol/L (5.0 mcg/dL) in combination with an elevated ACTH concentration more than twofold above the upper limit of the reference interval (10-60 pg/mL), and our patient fulfilled these two features. For confirmation, the corticotropin stimulation test (250 mcg) is currently considered the “gold standard” for the diagnosis of primary (but not secondary) adrenal insufficiency. It should be performed in most cases unless basal results are absolutely unequivocal [4].

The most common causes of PAI are autoimmune destruction of the adrenal cortex in adults and CAH in children [5-6]. These etiologies can be screened for by searching for 21-hydroxylase antibodies.

A CT scan of the adrenals may reveal evidence of adrenal infiltrative processes or metastases. Electrolyte abnormalities seen in AD include hyponatremia, hyperkalemia, mild hyperchloremic acidosis, hypoglycemia, normocytic anemia, coexisting pernicious anemia, and relative eosinophilia. However, none of these features were seen in our patient.

AD treatment includes hydrocortisone (15-25 mg) in two or three divided oral doses per day. As an alternative to hydrocortisone, prednisolone (3-5 mg/d), administered orally once or twice daily, is suggested, especially for patients with reduced compliance.

Monitoring of glucocorticoid replacement is conducted through clinical assessment, including body weight, postural blood pressure, energy levels, and signs of frank glucocorticoid excess. Aldosterone deficiency is confirmed either by clinical features or serum level. Although our patient exhibited neither clinical features of mineralocorticoid deficiency (such as salt craving, postural hypotension) nor had serum aldosterone levels assessed, the recommendation is to combine glucocorticoid and mineralocorticoid in individuals with AD. Patients with a confirmed aldosterone deficiency should receive mineralocorticoid replacement with fludrocortisone (the starting dose is 0.05-0.1 mg in adults) and should not restrict their salt intake. Monitoring mineralocorticoid replacement is primarily based on clinical assessment (including salt craving, postural hypotension, or edema) and blood electrolyte measurements [7].

Similar to our patient, the hyperpigmentation in AD typically begins to fade within several days and largely disappears after a few months of adequate glucocorticoid therapy.

## Conclusions

AD has a variety of clinical manifestations, but most of them are not specific. As in our case, which presented with sun-tan-like features, the hyperpigmentation in AD in our patient typically began to fade within several days and largely disappeared after a few months of adequate glucocorticoid therapy. We recommend increasing the threshold for investigating a patient suspected of having AD to avoid unnecessary complications.

## References

[1] Barthel A, Benker G, Berens K (2019). An update on Addison's disease. Exp Clin Endocrinol Diabetes.

[2] Jabbour SA (2003). Cutaneous manifestations of endocrine disorders: a guide for dermatologists. Am J Clin Dermatol.

[3] Lause M, Kamboj A, Fernandez Faith E (2017). Dermatologic manifestations of endocrine disorders. Transl Pediatr.

[4] Bornstein SR, Allolio B, Arlt W (2016). Diagnosis and treatment of primary adrenal insufficiency: an Endocrine Society clinical practice guideline. J Clin Endocrinol Metab.

[5] Zelissen PM, Bast EJ, Croughs RJ (1995). Associated autoimmunity in Addison's disease. J Autoimmun.

[6] Perry R, Kecha O, Paquette J, Huot C, Van Vliet G, Deal C (2005). Primary adrenal insufficiency in children: twenty years experience at the Sainte-Justine Hospital, Montreal. J Clin Endocrinol Metab.

[7] (2023). Primary adrenal insufficiency guideline resources. https://www.endocrine.org/clinical-practice-guidelines/primary-adrenal-insufficiency.

